# Impact of Aging on the Ovarian Extracellular Matrix and Derived 3D Scaffolds

**DOI:** 10.3390/nano12030345

**Published:** 2022-01-21

**Authors:** Georgia Pennarossa, Teresina De Iorio, Fulvio Gandolfi, Tiziana A. L. Brevini

**Affiliations:** 1 Laboratory of Biomedical Embryology, Department of Health, Animal Science and Food Safety and Center for Stem Cell Research, Università degli Studi di Milano, 20133 Milan, Italy; teresina.deiorio@unimi.it; 2 Laboratory of Biomedical Embryology, Department of Agricultural and Environmental Sciences—Production, Landscape, Agroenergy, Università degli Studi di Milano, 20133 Milan, Italy; fulvio.gandolfi@unimi.it

**Keywords:** reproductive aging, senescence, extracellular matrix, whole-ovary decellularization, ECM-based bio-scaffold, porcine

## Abstract

Advances in medical care, improvements in sanitation, and rising living standards contribute to increased life expectancy. Although this reflects positive human development, it also poses new challenges. Among these, reproductive aging is gradually becoming a key health issue because the age of menopause has remained constant at ~50 years, leading women to live longer in suboptimal endocrine conditions. An adequate understanding of ovarian senescence mechanisms is essential to prevent age-related diseases and to promote wellbeing, health, and longevity in women. We here analyze the impact of aging on the ovarian extracellular matrix (ECM), and we demonstrate significant changes in its composition and organization with collagen, glycosaminoglycans, and laminins significantly incremented, and elastin, as well as fibronectin, decreased. This is accompanied by a dynamic response in gene expression levels of the main ECM- and protease-related genes, indicating a direct impact of aging on the transcription machinery. Furthermore, in order to study the mechanisms driving aging and identify possible strategies to counteract ovarian tissue degeneration, we here described the successful production of a 3D ECM-based biological scaffold that preserves the structural modifications taking place in vivo and that represents a powerful high predictive in vitro model for reproductive aging and its prevention.

## 1. Introduction

In mammals, the female reproductive system is the first to show signs of physiological aging [1], with fertility decline and hormonal dysfunctions that can, in turn, affect overall health. This leads to multiple medical and psychosocial problems, such as osteoporosis, cardiovascular disease, autoimmune disorders, and depression [2,3]. Although medical care advances and rising living standards have contributed to increasing lifespan, the age of menopause has remained constant at the age of ~50 years, leading women to live longer in suboptimal endocrine conditions [4]. Reproductive aging has therefore gradually become a key health issue and an adequate understanding of its mechanisms is essential in order to prevent age-related diseases and to promote health and longevity in women [5].

Aging is generally described as a complex, multifaceted process, characterized by a progressive accumulation of macroscopic and microscopic modifications, accompanied by molecular and cellular damages that can affect organs, tissues, cells, and subcellular organelles, causing severe biological degenerations and a gradual loss of organ functions [6]. Recent studies also suggest that the non-cellular compartment of the tissues, the extracellular matrix (ECM), may play a key role in aging progression [7]. Indeed, during the last years, it has been clearly demonstrated that ECM contributes not only to physical scaffolding and structural support but also provides biochemical and biomechanical stimuli directly influencing cell behaviour [8]. Cells are indeed able to respond to physical and mechanical cues exerted by the surrounding environment modifying their own morphology, polarity, adhesion, migration, growth, gene expression, and functions [9,10,11,12,13]. However, cell ability to sense changes in ECM compliance (mechanosensation) and to transduce these stimuli into biochemical signals (mechanotransduction) are negatively affected by aging progression [6]. In addition, recent studies also suggested that aging may induce critical alterations in ECM composition and organization [14,15], resulting in biomechanical and biochemical ECM property modifications [6,16], that impact cell-to-matrix interactions as well as cell fate and behaviour.

Reproductive biology addressed growing interest in ovarian mechanobiology in the last decade and, in particular, the dynamic reciprocity that exists between ovarian cells and their microenvironment is currently under investigation [17,18]. In this contest, follicles and oocyte developmental quality have been demonstrated to be strongly influenced by biochemical cues [19] as well as by the physical ovarian microenvironment [20,21,22]. However, at present, only scattered information is available in the literature regarding aging mechanobiology of the reproductive tissues, where several aspects still need to be elucidated.

In the present study, we adopted the porcine as a model, based on its anatomical and physiological similarities with the human and we analyzed the impact of aging on ovarian ECM. In particular, we characterized and quantified the major ECM components, namely collagen, elastin, glycosaminoglycans, fibronectin and laminins, in young and aged ovaries. We analysed the ECM-related gene expression pattern in the two groups. Lastly, we generated 3D ECM-based biological scaffolds that preserve the age-specific ovarian milieu.

To the best of our knowledge, this is the first study reporting with great detail the changes taking place in the ECM of the senescent ovary, spanning from the structural remodeling to gene expression modifications. In addition, for the first time, the production of 3D ECM-based biological scaffolds that mimic the senescent ovarian microstructure is described. These represent an innovative tool to better characterize ovarian aging in a complex and physiologically relevant environment and will, in turn, allow to carry out in vitro studies aimed at the identification of novel therapeutic targets against ovarian senescence as well as potential anti-aging agents.

## 2. Materials and Methods

All reagents were purchased from Thermo Fisher Scientific (Milan, Italy) unless otherwise indicated.

### 2.1. Ovary Collection

There were 60 porcine ovaries collected at the local slaughterhouse and transported to the laboratory in sterile cold PBS. Organs from individuals ranging from 2 to 6 months of age were considered “young” (n = 30), while those from 3–5 years old animals were identified as “aged” (n = 30).

### 2.2. Histological Analysis

Young and aged ovaries were fixed in 10% buffered formalin for 24 h at room temperature, dehydrated in graded alcohols, cleared with xylene, and embedded in paraffin. Serial microtome sections of 5 µm in thickness were cut, dewaxed, rehydrated, and stained with hematoxylin and eosin (H&E, BioOptica, Milan, Italy), in order to evaluate the general morphological aspects.

### 2.3. Histochemical Analysis

Collagen, elastin, and GAGs were analyzed using Masson Trichrome (Bio-optica, Milan, Italy), Gomori’s aldehyde-fuchsin (Bio-optica), and Alcian blue (pH 2.5; Bio-optica, Milan, Italy) staining, respectively. Microtome sections (5 µm thick) were dewaxed, rehydrated, and stained following the manufacturer’s instructions. Samples were analyzed under an Eclipse E600 microscope (Nikon, Amsterdam, Netherlands), equipped with a digital camera (Nikon, Amsterdam, Netherlands). Pictures were acquired with NIS-Elements Software (Version 4.6; Nikon).

### 2.4. Immunohistochemical Analysis

Laminin subunit alpha 3 (LAMA3), laminin subunit beta 1 (LAMB1), and fibronectin 1 (FN1) were characterized by indirect immunohistochemistry using the Vectastain ABC Elite KIT (Burlingame, CA, USA). Laminin antigen retrieval was carried out in 10 mM sodium citrate buffer containing 0.05% Tween20 (pH 6) at sub-boiling temperature for 10 min. Tris-Base solution containing 0.05% Tween20 (pH 9) in pressure cooker for 5 min was used for FN1 unmasking. After cooling for 20 min, sections were rinsed and immersed in 3% H_2_O_2_ for 15 min to quench the endogenous peroxidase. Aspecific sites were blocked with Normal Serum for 30 min. Sections were then incubated with 1:100 LAMA3 (SantaCruz Biotechnology, CM6, California, USA), 1:500 LAMB1 (SantaCruz Biotechnology, LT3, California, USA), and 1:100 FN1 (Abcam, Cambridge, UK) antibodies diluted in 4% BSA in PBS, for 60 min in a humidified chamber. The appropriate biotinylated secondary antibody was added for 30 min. Lastly, tissues were stained with avidin-biotinylated horseradish peroxidase (HRP) complex for 30 min and with HRP substrate solution ImmPACT DAB substrate (Vector Laboratories, Burlingame, CA, USA). Sections were counterstained with Mayer’s hematoxylin, dehydrated, and permanently mounted (Bio-Optica, Milano, Italy). Secondary antibody controls were performed omitting the primary antibodies. All the procedures were carried out at room temperature unless otherwise indicated.

### 2.5. Stereological Analysis

Volume density (Vv) estimation of the ECM components was carried out on histochemical and immunohistochemical stained vertical sections, according to the general Delesse principle. In detail, the relative volume of each area of interest was calculated from the fractional area of the structure of interest (e.g., collagen) and the total area of the reference compartment (e.g., whole section). Systematic uniform random sampling was applied, and images were captured and superimposed with a point-count stereologic grid with equally distant test points. The points hitting the structure of interest and the respective reference compartment were counted, and the relative volume of each region of interest was then calculated from the respective quotient of points hitting these structures. Vv expressed as percentages was calculated as follows:

Vv _(analysed compartment, reference compartment)_ = [∑P_(analysed compartment)_/∑P_(reference compartment)_] × 100

where ∑P_(analysed compartment)_ is the number of points hitting the compartment under study, and ∑P_(reference compartment)_ is the number of points hitting the relevant structure.

### 2.6. ELISA Test

Quantifications of ECM components were carried out through ELISA tests using specific porcine ELISA kits (see Table 1). 400–500 mg of tissue were homogenized with Polytron (Kinematica AG, Malters, Switzerland), centrifuged at 3000 rpm for 20 min and supernatants were collected. Assays were carried out following the manufacturer’s instructions and, at the end of the procedures, collagen content was measured at 560 nm. Elastin, GAGs, LAMA3, LAMB1, and FN1 were quantified at 450 nm. Standard curves were constructed by plotting the mean absorbance (*y*-axis) against the protein concentration (*x*-axis) and the best fit line was determined by regression analyses. Absolute quantifications were then calculated.

### 2.7. Gene Expression Analysis

Young and aged ovary RNA was extracted using the TaqManGene Expression Cells to Ct kit (Applied Biosystems, Milan, Italy) and following the manufacturer’s instruction. DNase I was added to lysis solution at a 1:100 concentration. Quantitative PCR was performed on a CFX96 Real-Time PCR (Bio-Rad, Milan, Italy) using predesigned gene-specific primers and probe sets from TaqManGene Expression Assays (see Table 2 for the primer information). ACTB and GAPDH were used as internal reference genes. Target gene quantification was carried out with CFX Manager software 18450000 (Bio-Rad, Milan, Italy). Gene expression levels are reported with the highest expression set to 1 and the others relative to this.

### 2.8. Whole-Ovary Decellularization

Young and aged whole-ovaries were decellularized according to the protocol previously developed in our laboratory [23,24,25]. Briefly, the entire organs were frozen at –80 °C for at least 24 h. They were then thawed at 37 °C in a water bath for 30 min, followed by an incubation with 0.5% sodium dodecyl sulfate (SDS; Bio-Rad, Milan, Italy) in deionized water (dd-H_2_O) for 3 h. Ovaries were then treated overnight with 1% Triton X-100 (Sigma, Milan, Italy) in dd-H_2_O, extensively washed in dd-H_2_O for 9 h, and, subsequently, immersed in 2% deoxycholate in dd-H_2_O (Sigma, Milan, Italy) for 12 h. Lastly, decellularized whole-ovaries were washed in dd-H_2_O for 6 h, with changes every 2 h. All steps were carried out at room temperature using an orbital shaker at 200 rpm.

At the end of the decellularization process, age-specific ovarian ECM-based scaffolds obtained were subjected to histological (please see 2.2. section) and histochemical analyses (please see 2.3. section), ELISA tests (as described above in 2.4. section), cell density analysis, and DNA quantification studies.

### 2.9. Cell Density

Efficient cell removal from the decellularized whole-ovaries was assessed by staining serial microtome sections with 40, 6-diamidino-2-phenylindole (DAPI). Cell number was counted in 15 DAPI-stained tissue sections obtained from each decellularized ovarian scaffold. A total of 5 randomly selected fields at ×100 total magnifications were analysed for each section. Images were acquired with constant exposure parameters and analysed using the Automated Cell Counter tool of ImageJ software [26] following the provider’s instructions. Briefly, 8-bit images were generated, and threshold adjustments were applied. Subsequently, in order to remove the background and highlight the area occupied by nuclei, the images were segmented with a thresholding algorithm. The data obtained were then transformed in binary form and size and circularity parameters were set in order to automatically count nuclei. Cell density was expressed per mm^2^ of tissue.

### 2.10. DNA Quantification

Ten fragments, ranging from 10 to 24 mg, were cut from each decellularized ovarian ECM-based scaffold and their weights were annotated. Genomic DNA was then extracted from each fragment with the PureLink^®^ Genomic DNA Kit, following the manufacturer’s instructions. The DNA concentration was measured with NanoDrop 8000 and normalized against the fragment weights previously recorded.

### 2.11. Statistical Analysis

Statistical analyses were performed using one-way and two-way ANOVA (SPSS 19.1; IBM). Data are presented as the mean ± the standard error of the mean (SEM). Differences of *p* ≤ 0.05 were considered significant and were indicated with different superscripts.

## 3. Results

### 3.1. Macroscopic and Microscopic Analysis of Young and Aged Ovaries

**Figure 1:**
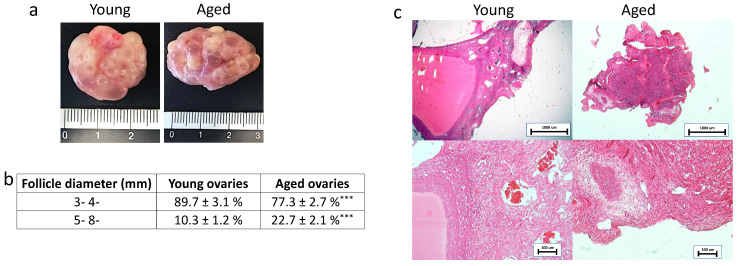
Macroscopic and histological evaluations of young and aged ovaries. (**a**) Representative macroscopic images illustrating young (left panel) and aged ovaries (right panels); (**b**) 3–4 mm and 5–8 mm follicle rate observed in young and aged ovaries, *** *p* < 0.001; (**c**) H & E staining showed the typical ovarian tissue architecture with primordial, growing and antral follicles, ovarian stroma and vasculature in both young and aged tissues. Scale bars = 100 μm.

Macroscopic evaluation revealed the typical morphology of young and adult ovaries with numerous follicles ranging from 3- to 8- mm in diameter (Figure 1a). As expected, smaller follicle sizes, 3- 4- mm, were more abundant than larger ones (5- 8- mm) in both young and aged ovaries. However, the rate of 3- 4-mm follicles was higher in young ovaries, while a higher proportion of 5- 8-mm follicles was found in aged ovaries (Figure 1b).

In order to evaluate the general morphological aspect of young and aged ovaries, H&E staining was carried out. The analyzed tissues showed the typical ovarian tissue architecture consisting of primordial, growing, and antral follicles, ovarian stroma, and vasculature in both young and aged tissues. However, aged ovaries displayed a denser and more compact stromal compartment compared to the young ones (Figure 1c).

### 3.2. Aging Effects on Ovarian Extracellular Matrix Composition

In order to characterize the major ECM components, we selected specific histological- and immuno-staining. In addition, a quantitative evaluation was carried out through stereological analysis and ELISA tests.

**Figure 2:**
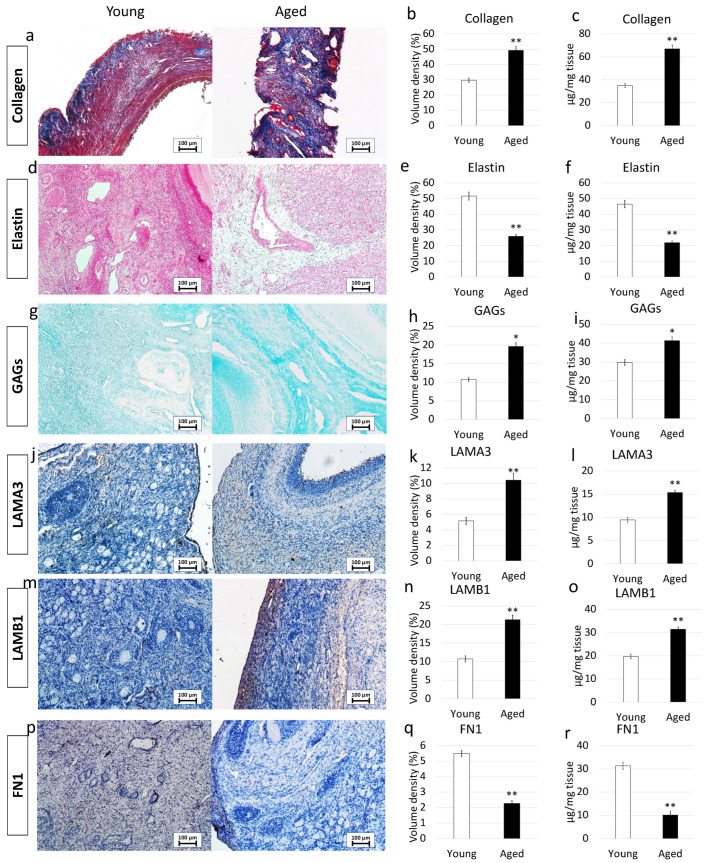
Histochemical and immunohistochemical analysis, stereological quantification, and ELISA tests in young and aged ovaries. (**a**) Masson’s trichrome staining showed the presence of collagen (blue) and elastic (magenta) fibers in both young and aged ovaries. Scale bars = 100 μm; (**b**) Stereological analysis demonstrated a significant increase of collagen fibers in aged ovaries compared to the young ones. Data are expressed as the mean. Error bars represent the standard error of the mean (SEM), ** *p* < 0.01; (**c**) ELISA quantifications indicated a significant increase of the collagen content in aged ovaries compared to the young ones. Data are expressed as the mean. Error bars represent the standard error of the mean (SEM), ** *p* < 0.01; (**d**) Gomori’s Aldehyde Fuchsin staining revealed the presence of elastic fibers in both young and aged ovaries. Scale bars = 100 μm; (**e**) Stereological analysis displayed a significant decrease of elastic fibers in aged ovaries compared to the young ones. Data are expressed as the mean. Error bars represent the standard error of the mean (SEM), ** *p* < 0.01; (**f**) ELISA quantifications confirmed a significant decrease of the elastin content in aged ovaries compared to the young ones. Data are expressed as the mean. Error bars represent the standard error of the mean (SEM), ** *p* < 0.01; (**g**) Alcian blue staining showed the presence of GAGs in both young and aged ovaries. Scale bars = 100 μm; (**h**) Stereological analysis indicated a significant increase of GAGs in aged ovaries compared to the young ones. Data are expressed as the mean. Error bars represent the standard error of the mean (SEM), * *p* < 0.05; (**i**) ELISA quantifications confirmed a significant increase of the GAG content in aged ovaries compared to the young ones. Data are expressed as the mean. Error bars represent the standard error of the mean (SEM), * *p* < 0.05; (**j**) Immunohistochemical staining of young and aged ovaries for LAMA3. Scale bars = 100 μm; (**k**) Stereological analysis demonstrated a significant increase of LAMA3 in aged ovaries compared to the young ones. Data are expressed as the mean. Error bars represent the standard error of the mean (SEM), ** *p* < 0.01; (**l**) ELISA quantifications showed a significant increase of the LAMA3 content in aged ovaries compared to the young ones. Data are expressed as the mean. Error bars represent the standard error of the mean (SEM), ** *p* < 0.01; (**m**) Immunohistochemical staining of young and aged ovaries for LAMB1. Scale bars = 100 μm; (**n**) Stereological analysis revealed a significant increase of LAMB1 in aged ovaries compared to the young ones. Data are expressed as the mean. Error bars represent the standard error of the mean (SEM), ** *p* < 0.01; (**o**) ELISA quantifications displayed a significant increase of the LAMB1 content in aged ovaries compared to the young ones. Data are expressed as the mean. Error bars represent the standard error of the mean (SEM), ** *p* < 0.01; (**p**) Immunohistochemical staining of young and aged ovaries for FN1. Scale bars = 100 μm; (**q**) Stereological analysis indicated a significant decrease in FN1 content in aged ovaries compared to the young ones. Data are expressed as the mean. Error bars represent the standard error of the mean (SEM), ** *p* < 0.01; (**r**) ELISA quantifications showed a significant increase of the LAMB1 content in aged ovaries compared to the young ones. Data are expressed as the mean. Error bars represent the standard error of the mean (SEM), ** *p* < 0.01.

More in detail, Masson trichrome showed a robust blue staining in aged ovaries, indicating an increment in collagen content (Figure 2a). This was also confirmed by stereological analysis (Figure 2b) as well as ELISA quantifications (Figure 2c) that demonstrated significantly higher concentrations of collagen fibers in aged ovaries compared to their young counterpart.

Elastin specific staining Gomori’s Aldehyde Fuchsin demonstrated a decrease in elastic fiber content in aged samples (Figure 2d). This is further supported by stereological analysis (Figure 2e) and ELISA quantifications (Figure 2f), which indicated a significant decrease in elastic fiber volume density in aged ovaries.

In contrast, Alcian blu staining (Figure 2g) and stereological analysis (Figure 2h) demonstrated an increased quantity of GAGs in aged ovaries compared to the young ones. This was also confirmed by ELISA tests that indicated a significantly higher concentration of GAG content in aged ovaries (Figure 2i).

Immunohistochemical characterization demonstrated a higher amount of LAMA3 and LAMB1 in aged ovaries compared to the young group (Figure 2j,m). This was confirmed by stereological (Figure 2k,n) and ELISA quantifications (Figure 2l,o). On the other hand, FN1 staining (Figure 2p), stereological analysis (Figure 2q) and ELISA tests (Figure 2r) indicated a decreased fibronectin quantity in aged ovaries.

### 3.3. Aging Effects on Extracellular Matrix-Related Gene Expression

**Figure 3:**
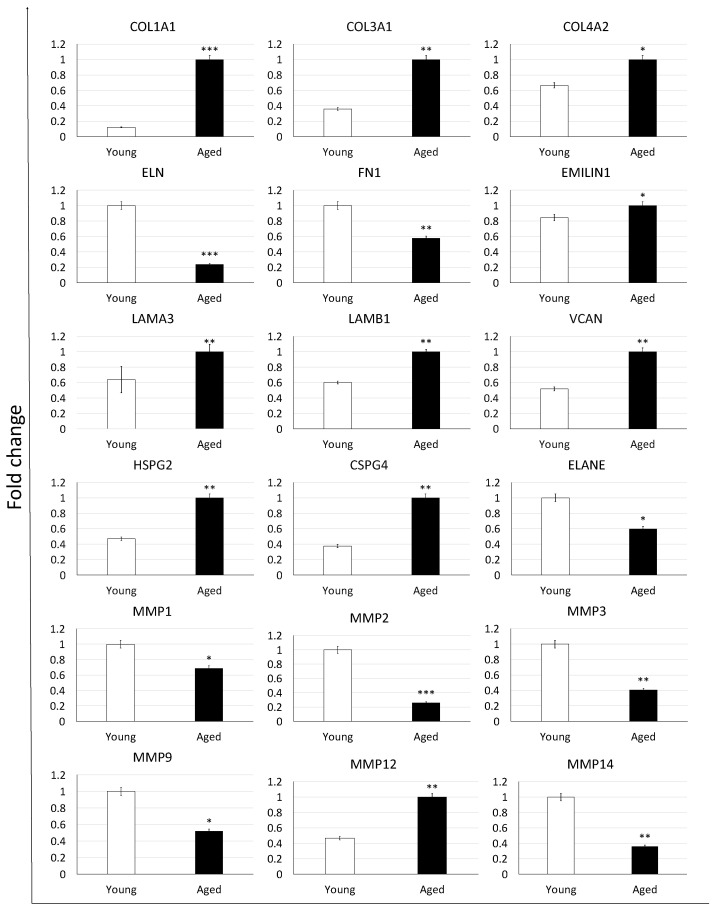
Gene expression changes of extracellular matrix-related genes in young (white bars) and aged ovaries (black bars). Expression levels of collagens (COL1A1, COL3A1, COL4A2), elastin (ELN), fibronectin (FN1), glycoprotein (EMILIN1), laminins (LAMA3, LAMB1), proteoglycans (VCAN, HSPG2, CSPG4), and proteases (ELANE, MMP1, MMP2, MMP3, MMP9, MMP12, and MMP14). Data are expressed as the mean. Error bars represent the standard error of the mean (SEM), * *p* < 0.05, ** *p* < 0.01, *** *p* < 0.001.

To confirm the morphological data revealing ECM composition changes with aging progression, we profiled expression patterns and levels of ECM-related genes (Figure 3). While no significant modifications in expression patterns were detected, we could assess a very dynamic response in the transcription levels of the genes considered. In particular, we were able to demonstrate significantly increased expression levels of collagens (COL1A1, COL3A1, COL4A2), EMILIN1 glycoprotein, laminins (LAMA3, LAMB1), and proteoglycans (VCAN, HSPG2, CSPG4) in aged ovaries compared to the young ones. In contrast, down regulation of elastin (ELN)and fibronectin (FN1) accompanied the aging process which also caused a decreased expression of various proteases that hydrolyze the different ECM components, namely ELANE, MMP1, MMP2, MMP3, MMP9, and MMP14. Interestingly, the matrix metalloproteinase involved in elastin degradation, the MMP12, was upregulated in aged ovaries.

### 3.4. Whole-Ovary Decellularization Protocol Successfully Removes Cell Compartment and Preserves Age-Specific Ovarian Architecture

**Figure 4:**
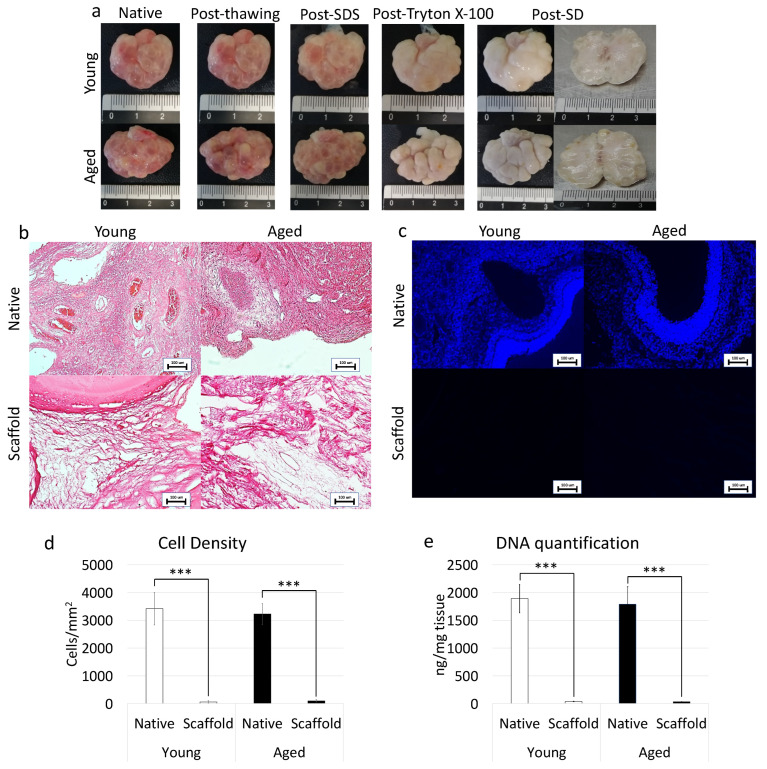
Macroscopic and microscopic evaluations of young and aged ECM-based scaffolds, cell density, and DNA quantification. (**a**) Chronological macroscopic images illustrating the decellularization process in young (upper panel) and aged ovaries (lower panels). Native and decellularized organs displayed comparable shapes and homogeneity, while their color turns from red to white along the process; (**b**) H&E staining showed the presence of both basophilic (cell nuclei) and eosinophilic (cell cytoplasm and ECM) staining in the control tissues (Young Native and Aged Native), while cell nuclei and the related basophilic staining were absent in the decellularized ECM-based scaffolds (Young Scaffold and Aged Scaffold). Scale bars = 100 μm; (**c**) DAPI staining displayed the presence of nuclei in native ovaries (Young Native and Aged Native), which disappeared after the decellularization process (Young Scaffold and Aged Scaffold). Scale bars = 100 μm; (**d**) Cell density demonstrated a significantly lower number of nuclei in both young and aged decellularized ECM-based scaffolds (Scaffold) compared to the untreated tissues (Native). Data are expressed as the mean. Error bars represent the standard error of the mean (SEM), *** *p* < 0.001; (**e**) DNA quantification analysis showed a significant decrease in the DNA content of young and aged decellularized ECM-based scaffolds (Scaffold) compared to the native tissue (Native). Data are expressed as the mean. Error bars represent the standard error of the mean (SEM), *** *p* < 0.001.

In order to assess the efficacy of the whole-organ decellularization protocol, macroscopic evaluations were carried out along the decellularization process. The results obtained demonstrated that both young and aged ovaries preserved their original shape and homogeneity, without any deformation and maintained the age-related morphological differences already detectable in the native organs. In addition, comparable changes in whole organ color, turning from red to white, were visible in both the experimental groups (Figure 4a).

In addition, H&E staining was performed to evaluate the general morphological aspect of the decellularized whole-ovaries. The data obtained demonstrated the successful removal of the cellular compartment and the maintenance of general structure in both young and aged decellularized ovaries. More in detail, the basophilic staining, clearly visible in the native tissues, was absent after the decellularization processes in all samples analyzed, regardless of age (Figure 4b). In agreement with this, DAPI staining (Figure 4c) and cell density evaluations (Figure 4d) showed a significantly lower number of cell nuclei in both young and aged decellularized ovaries compared to the untreated tissues. This is also confirmed by DNA quantification studies indicating a 98.07% and a 98.22% decrease in young and aged decellularized ovaries, respectively. In particular, a content of 36.36 ± 4.54 ng DNA/mg and 31.75 ± 2.29 ng DNA/mg of tissue was measured in young and aged decellularized tissues vs. 1890.28 ± 257.37 ng DNA/mg and 1789.96 ± 313.55 ng DNA/mg of tissue in young and aged native controls (Figure 4e).

### 3.5. Whole-Ovary Decellularization Protocol Maintains Unaltered Aged-Specific Extracellular Matrix Composition

#### 3.5.1. Histochemical Characterization

Maintenance of age-specific ECM composition and organization after decellularization was investigated using specific histochemical staining for collagen, elastin, and GAGs.

**Figure 5:**
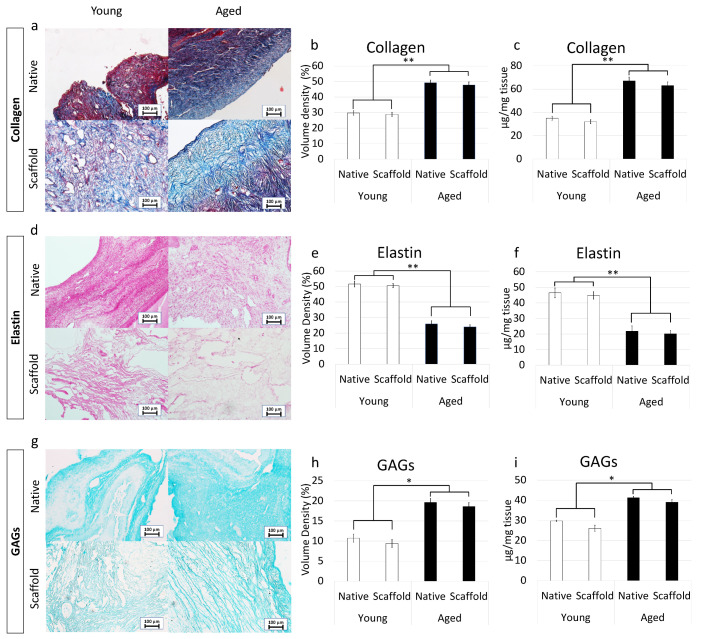
ECM microarchitecture and composition in young and aged ECM-based scaffolds. (**a**) Masson’s Trichrome staining showed the persistence of collagen fibers (blue) and their comparable distribution between native (Native) and decellularized ovaries (Scaffold) belonging to the same age. (**b**,**c**) Stereological (**b**) and ELISA quantifications (**c**) demonstrated no significant differences between native ovaries (Native) and the decellularized ECM-based scaffolds (Scaffold) in collagen content. Data are expressed as the mean. Error bars represent the standard error of the mean (SEM), ** *p* < 0.01; (**d**) Gomori’s aldehyde-fuchsin staining indicated that ECM-based scaffolds (Scaffold) retained the elastic fibers scattered throughout the decellularized ovary, similar to what was visible in untreated young and aged ovaries (Native). (**e**,**f**) Stereological (**e**) and ELISA quantifications (**f**) confirmed no significant differences between native ovaries (Native) and the decellularized ECM-based scaffolds (Scaffold) in elastin content. Data are expressed as the mean. Error bars represent the standard error of the mean (SEM), ** *p* < 0.01; (**g**) Alcian blue staining revealed GAG retention in young and aged decellularized scaffolds (Scaffold). Scale bars = 100 μm; (**h**,**i**) Stereological (**h**) and ELISA quantifications (**i**) demonstrated no significant differences between native ovaries (Native) and the decellularized ECM-based scaffolds (Scaffold) in GAG content. Data are expressed as the mean. Error bars represent the standard error of the mean (SEM), * *p* < 0.05.

More in detail, Masson trichrome staining showed the persistence of collagen fibers in both young and aged decellularized ovaries, with comparable distribution between decellularized and native tissues belonging to the same age. In agreement with this, at the end of the decellularization processes, collagen fibers maintained the age-specific organization detected in the native tissues (Figure 5a). These morphological observations were also confirmed by stereological analysis, where no significant differences were detected between native and decellularized tissues of the same age (Figure 5b). In agreement with this, the age-related differences originally identified in the native tissues were maintained after the decellularization process (Figure 5b). These data were further supported by ELISA tests indicating comparable collagen content in young native and decellularized ovaries (35.1 ± 4.1 µg/mg and 32.3 ± 3.9 µg/mg of tissue) as well as in aged native and decellularized tissues (67.1 ± 9.1 µg/mg and 63.2 ± 8.9 µg/mg of tissue; Figure 5c).

Gomori’s aldehyde-fuchsin staining demonstrated that decellularized tissues preserved elastic fibers scattered throughout the ovary, regardless of age. Consistently, decellularized young and aged ovaries displayed the same differences observed in the native organs (Figure 5d). This was further supported by stereological analysis displaying no significant differences between young decellularized and native tissue as well as between aged decellularized and native ovaries (Figure 5e). Similarly, elastin quantification studies showed a comparable amount of elastin before and after the decellularization process in young (46.5 ± 3.12 µg/mg and 44.85 ± 2.32 µg/mg) and aged (21.87 ± 3.34 µg/mg and 20.09 ± 2.01 µg/mg) tissues (Figure 5f).

Alcian blue staining revealed GAG retention in both young and aged tissues, preserving the age-specific distributions visible in the native ovaries (Figure 5g). These morphological observations were confirmed by both stereological analysis (Figure 5h) and ELISA quantification studies (Figure 5i), which, as seen for the other ECM components, displayed no significant GAG reduction after the decellularization.

#### 3.5.2. Immunohistochemical Characterization

**Figure 6:**
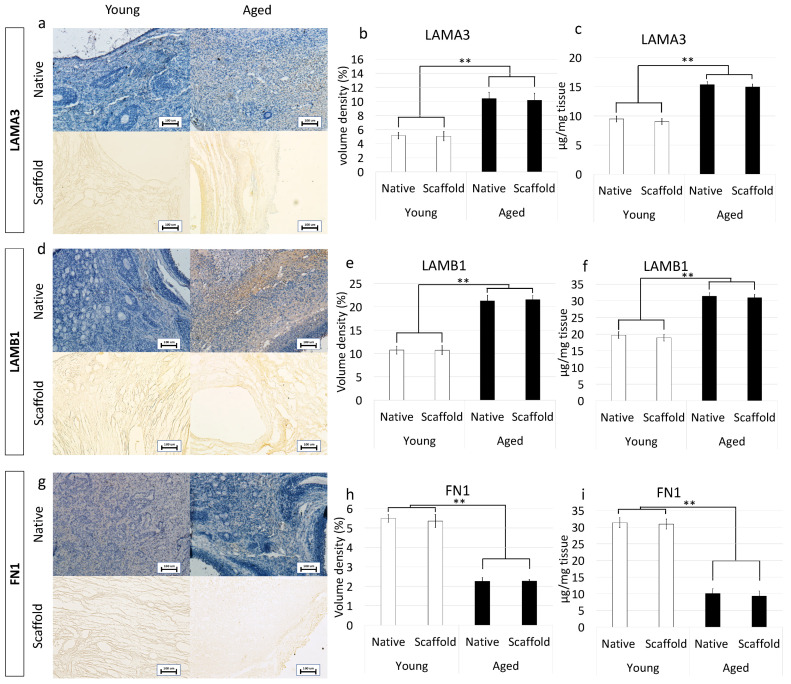
Immunohistochemical characterizations of young and aged ECM-based scaffolds. (**a**) LAMA3 immunostaining displayed the persistence of the fibers after decellularization in both young and aged tissues; Stereological analysis (**b**) and ELISA quantifications **(c)** demonstrated no significant differences between native (Native) and decellularized (Scaffold) ovaries in LAMA3 content. Data are expressed as the mean. Error bars represent the standard error of the mean (SEM), ** *p* < 0.01; (**d**) LAMB1 immunostaining showed the maintenance of the fibers at the end of the process in both young and aged organs; Stereological analysis (**e**) and ELISA tests (**f**) indicated no significant differences between native (Native) and decellularized tissues (Scaffold) in LAMB1 content. Data are expressed as the mean. Error bars represent the standard error of the mean (SEM), ** *p* < 0.01; (**g**) FN1 immunostaining revealed fiber retainment in both young and aged decellularized tissues; Stereological (**h**) and ELISA analysis (**i**) showed no significant differences between native (Native) and decellularized samples (Scaffold) in FN1 content. Data are expressed as the mean. Error bars represent the standard error of the mean (SEM), ** *p* < 0.01.

Preservation of LAMA3, LAMB1, and FN1 in the produced bio-scaffold was investigated using immunohistochemical analysis. The results obtained demonstrated that decellularized ovaries maintained unaltered distribution of LAMA3, LAMB1, and FN1 compared to the untreated native tissues (Figure 6a,d,g). This was confirmed by stereological analysis indicating significantly lower volume densities of LAMA3 (Figure 6b) and LAMB1 (Figure 6e) in both native and decellularized young tissues compared to their aged counterparts. In contrast, FN1 content was higher in young samples as displayed by immunohistochemical staining (Figure 6g) and stereological analysis (Figure 6h). These evaluations were also supported by ELISA quantifications demonstrating a higher amount of LAMA3 (Figure 6c) and LAMB1 (Figure 6f) in aged native and decellularized tissues and lower content of FN1 (Figure 6i).

## 4. Discussion

In the present work, we characterize the impact of aging on ovarian ECM and report the possibility to successfully apply a whole-organ decellularization technique to produce for the first time ECM-based scaffolds that preserve the structural modifications taking place in the senescent ovary.

Macroscopic and microscopic evaluations of aged ovaries reveal distinctive morphological features when compared to the young counterparts. A significantly lower number of small follicles (3–4 mm) and a higher number of larger ones (5–8 mm) are scored in aged ovaries. This appears to be strictly correlated with the age of the animals and as previously described in several mammalian species, ranging from mouse [27] and cow [28] to the human [29] is likely to be associated with age-related infertility. A fundamental difference of aged ECM structure is also represented by the presence of a denser and more compact stromal compartment, which suggests the possibility of distinct matrix re-arrangements taking place during the aging process. This hypothesis is further confirmed by the histochemical results that indicate, both with Masson trichrome staining and stereological analysis, an increase in collagen fibers. A significantly higher quantity of collagen is also demonstrated by ELISA tests, comparing aged ovaries vs. young ones. These data are in agreement with recent studies, carried out on reproductively old mouse models, reporting a quantitative increase in ovarian stiffness due to collagen deposition [1,7,22]. It is interesting to remember that accumulation of collagen is an important event contributing to tissue fibrosis that is usually identified as one of the most common aging-associated hallmarks, exhibited by a wide range of tissues, including liver [30,31], heart [14,32], and lung [15], and that, in turn, induces tissue architecture disruption and affects organ biomechanics and functions [33]. In addition, it has been demonstrated that stiffness increase may also be correlated with age-associated tumor development, facilitating ovarian cancer cell proliferation and aggregation [34]. Senescence-specific increment in matrix rigidity has been also accounted for alterations in collagen-to-elastin ratio and/or a general decrease in elastin content [35,36]. This fully fits with the results presented here, showing a significant decrease of elastic fibers in aged ovaries, both by Gomori’s aldehyde fuchsin staining and stereological analysis and clearly displaying a significantly lower number of elastic fibers in aged tissues compared to the young ones. These observations are consistent with the dramatic fall in elastin levels detected in menopausal women perifollicular ECM [20] and are well in agreement with the unbalanced collagen/elastin ratio here demonstrated with ELISA quantification studies in senescent ovaries. Altogether, this suggests that similar to that which was described in other tissues [37,38,39], ovarian aging can be associated with a progressive elasticity reduction and, therefore, with a general decrement in mechanical resilience and durability. However, the increase in GAG content, demonstrated in the present study, indicates that these molecules may act to buffer the altered collagen/elastin balance, therefore, suggesting the existence of compensative mechanisms, possibly mitigating the decrease in tissue elasticity.

Interestingly enough, the changes described above are accompanied by a significant increase of the laminins LAMA3 and LAMB1 in aged samples. Although only few data are available on laminin-matrix deposition mechanisms, the signaling pathways involved, and the role in specifying cell behaviors [40], it must be noted that several studies reported high laminin contents in senescent cells isolated from fibrotic tissues, including aorta [41], lung [42], and skin [43]. We may therefore speculate that laminin deposition may be correlated and/or contribute to age-related ECM modifications [44] and further studies are needed in other to better clarify these aspects and the related mechanisms. The increase in laminins is also paralleled by a significant reduction in FN1 content. This is in line with Schnabl et al., and Krizhanovsky et al., which reported a reduction in FN expression levels in liver fibrosis [45,46]. On the other hand, an opposite trend was described by other authors that showed FN transcription upregulation in different organs [44,47,48]. We have no clear explanation for such discrepancies, but we may hypothesize that the different results obtained may be related to cell type-specific differences and/or tissue-specific responses.

A very dynamic variation in gene transcription levels, closely reflecting the morphological data described above, accompanies ECM reorganization in the senescent ovary, with higher expression levels of collagens (COL1A1, COL3A1, COL4A2), glycoprotein (EMILIN1), laminins (LAMA3, LAMB1), proteoglycans (VCAN, HSPG2, CSPG4), and downregulation of elastin (ELN) and fibronectin (FN1). Interestingly, all the protease-related genes analyzed, namely ELANE, MMP1, MMP2, MMP3, MMP9, and MMP14, show significantly lower expression levels in aged ovaries, suggesting that ECM degradation and remodeling activities may decrease during senescence, while protein deposition mechanisms are kept active, resulting in fiber accumulation. In contrast to all the protease analyzed, MMP12 transcription is significantly increased in senescent ovaries. Since this gene encodes for a matrix metalloproteinase involved in elastin degradation, its upregulation may well explain the significant reduction in elastic fiber content described above. In our understanding, the molecular results here reported demonstrate a fine-tuning in gene expression activity that orchestrates ECM composition changes characterizing the aging process and that suggests a direct impact of senescence on the transcription machinery.

When a whole-organ decellularization protocol, previously developed in our laboratory, [23,24,25] was applied to aged ovaries, we were able to derive 3D ECM-based biological scaffolds that represent a viable alternative to the use of synthetic matrix such as hydrogels [49]. The decellularized scaffolds preserve all the changes that are described above and that are distinctive of the senescent organ. Both histochemical and immunohistochemical staining demonstrate indeed the preservation and the quantities of collagen, elastin, GAGs, laminins, and fibronectin detected in aged native tissues, thus basically confirming the maintenance of the original aged organ. These observations are coherent with our previous studies in which scanning electron microscopy analysis, as well as histological staining, demonstrated that the decellularization process here adopted guaranteed an efficient cell removal paralleled by the maintenance of microarchitecture integrity with intact ECM framework and well-connected and oriented fibers [23,24]. The results obtained also suggest that the generated scaffold maintains an intact basement membrane complex after the decellularization process, which has been previously demonstrated to play a key role in modulating in vitro cell growth patterns [50]. This is, to our knowledge, the first attempt to create a biological matrix that mimics the senescent ovarian microstructure and offers an exciting opportunity to better characterize ovarian aging in vitro in a complex and physiologically relevant environment.

## 5. Conclusions

Significant changes take place in the aged ovarian ECM. These include altered concentrations of the main structural molecules, such as collagen, elastin, laminins, and fibronectin, that lead to tissue architecture remodeling, increment of fibrosity, and reduced elasticity. Interestingly, age-related modifications are not only the results of post-transcriptional processes but rather have a direct impact on gene expression.

The typical aging phenotype is preserved after decellularization, with the creation of an ECM-based scaffold that mimics the senescent ovarian microstructure and offers an exciting opportunity to better characterize ovarian aging in vitro in a complex and physiologically relevant environment. This, in turn, constitutes an alternative high predictive in vitro model for the research in the aging field, allowing for a reduction of the number of experimental animals used. At the same time, the bio-scaffold will in due time implement the production of an “aging ovary in a dish” model to further study the mechanisms driving aging and, when coupled with non-cytotoxic nanovehicles [51], may lead to the identification of possible strategies to counteract tissue degeneration.

## Figures and Tables

**Table 1:** ELISA kits used to quantify the different ECM components.

Protein	Description	Cat.N.
Collagen	Total Collagen Assay Kit (Bio-Techne SRL, Milano, Italy)	NBP2-59748
Elastin	Porcine Elastin, ELN ELISA Kit (BT Lab, Shanghai, China)	E0504Po-96T
GAGs	Porcine Glycosaminoglycans, GAGs ELISA Kit (BT Lab, Shanghai, China)	E0503Po-96T
LAMA3	Porcine Epiligrin ELISA Kit (MyBioSource, San Diego, CA, USA)	MBS019792
LAMB1	Porcine Laminin subunit beta-1 (LAMB1) ELISA Kit (MyBioSource, San Diego, CA, USA)	MBS2613645
FN1	Fibronectin (FN), ELISA Kit (MyBioSource, San Diego, CA, USA)	MBS2700851

**Table 2:** List of primers used for quantitative PCR analysis.

Gene	Description	Cat.N.
ACTB	Actin, beta	Ss03376563_uH
GAPDH	Glyceraldehyde-3-phosphate dehydrogenase	Ss03375629_u1
COL1A1	Collagen type I alpha 1 chain	Ss03373341_g1
COL3A1	Collagen type III alpha 1 chain	Ss04323772_g1
COL4A2	Collagen type IV alpha 2 chain	Ss06936852_mH
ELN	Elastin	Ss04955050_m1
FN1	Fibronectin 1	Ss03373883_m1
EMILIN1	Elastin microfibril interfacer 1	Ss06869485_m1
LAMA3	Laminin subunit alpha 3	Ss06874585_g1
LAMB1	Laminin subunit beta 1	Ss03375563_u1
VCAN	Versican	Ss04323138_m1
HSPG2	Heparan sulfate proteoglycan 2	Ss06878501_m1
CSPG4	Chondroitin sulfate proteoglycan 4	Ss03374044_m1
ELANE	Elastase, neutrophil expressed	Ss06915973_gH
MMP1	Matrix metallopeptidase 1	Ss04245657_g1
MMP2	Matrix metallopeptidase 2	Ss04955620_m1
MMP3	Matrix metallopeptidase 3	Ss03375473_u1
MMP9	Matrix metallopeptidase 9	Ss03392100_m1
MMP12	Matrix metallopeptidase 12	Ss03386225_u1
MMP14	Matrix metallopeptidase 14	Ss03394427_m1

## Data Availability

The data presented in this study are available on request from the corresponding author.
